# Theoretical and clinical insights into the psychological mechanisms and active ingredients of emotional freedom techniques

**DOI:** 10.3389/fpsyg.2026.1672132

**Published:** 2026-04-07

**Authors:** R. Schwarz, P. Stapleton

**Affiliations:** 1 Private Practitioner, Philadelphia, PA, United States; 2 Faculty of Society and Design, Bond University, Gold Coast, QLD, Australia

**Keywords:** emotional freedom techniques (EFT), psychotherapy, psychological intervention, active ingredients, mechanisms of action, dysregulation, emotional avoidance, memory reconsolidation

## Abstract

Clinical Emotional Freedom Techniques (EFT) is an integrative mind–body therapy increasingly utilized for trauma and stress-related conditions. This article is a theoretical proposal rather than a systematic review; its purpose is to synthesize existing evidence and clinical insights to outline a conceptual framework of active ingredients and mechanisms underlying EFT’s therapeutic efficacy. Active ingredients include acupoint stimulation, exposure within the window of tolerance, integration of self-compassion and acceptance, leveraging demand characteristics and positive expectancy, shifting and titrating the focus of awareness, and the strategic use of the in-session processing. These elements facilitate four key mechanisms: (1) reduction of physiological and emotional dysregulation, (2) reduction of emotional avoidance, (3) cognitive restructuring, and (4) memory reconsolidation. Clinical EFT’s emphasis on in-session processing and body engagement, particularly meridian point tapping, addresses autonomic nervous system dysregulation and promotes a positive relationship with one’s physical self. By integrating these active ingredients and mechanisms, Clinical EFT offers a comprehensive approach to trauma treatment that addresses both psychological and physiological dimensions of distress, potentially improving treatment adherence and promoting lasting emotional and cognitive change.

## Introduction

Over the last 20 years, energy psychology (EP) approaches have evolved from having minimal research support to being the subject of over 200 studies published in English language refereed journals. Remarkably, 99% of those studies report statistically significant positive outcomes (Church et al., 2022). Among the 200+ studies, 100+ have been randomized controlled trials and 95+ have been outcome studies. Notably, most of this research has been conducted in the past decade. The majority of these studies have concentrated on two acupoint tapping approaches: Clinical Emotional Freedom Techniques (EFT) and Thought Field Therapy (TFT). The focus of this paper is Clinical EFT that traces to the original EFT protocol as developed by Craig (Craig and Fowlie, 1995). The method integrates cognitive, somatic, and exposure elements, including clinical protocols, coaching strategies, and a variety of self-help approaches. Within this category, Clinical EFT refers to the standardized, manualized protocol developed through systematic research and codified in published guidelines (Church, 2018; Stapleton and Janzen, 2025). Clinical EFT specifies core components such as the Basic Recipe, setup phrases, and structured techniques such as Tell the Story and The (Silent) Movie Technique, and it incorporates principles of trauma-informed care, titrated exposure, and therapist-guided regulation. Importantly, the majority of randomized controlled trials and international guidelines (e.g., cost-effectiveness analyses; Mavranezouli et al., 2020a, 2020b) are based on Clinical EFT rather than informal or self-help variants. This distinction is critical because informal EFT may omit key active ingredients—such as strategic titration and therapist utilization—that underpin mechanisms like memory reconsolidation and autonomic regulation.

This article is a theoretical proposal rather than an empirical review. Its primary aim is to synthesize existing research and clinical insights to articulate a conceptual framework for understanding the active ingredients and mechanisms underlying Clinical EFT. While the discussion draws on diverse strands of evidence—including psychological, somatic, and neurophysiological perspectives—our intent is not to provide a comprehensive systematic review, but to propose an integrative model that can guide future research and clinical refinement. The paper is structured around three core objectives: (1) delineating the active ingredients embedded within Clinical EFT protocols, (2) mapping these ingredients to hypothesised mechanisms of change, and (3) identifying implications for research and practice. By clarifying these theoretical foundations, we aim to advance dialogue on EFT’s clinical application and mechanistic plausibility.

Over the past 20 years, EP approaches have sparked significant controversy. Clinicians have claimed unusual effectiveness while some academics and others have been skeptical, even hostile, toward these methods. Initially, critics rightly pointed out the lack of research supporting EP. However, as research began to validate clinical claims, criticisms shifted to the methodologies used. These criticisms were valid to some extent. Yet, as methodologies improved, the positive results and consistency of EP endured, becoming broader, stronger, and clearer.

## Evidence to date (a brief synopsis)

Over the last 20 years, energy psychology (EP) approaches have evolved from having minimal research support to being the subject of over 200 studies published in refereed journals. Remarkably, 99% of those studies report significant positive outcomes (Church et al., 2022). Among the 200+ studies, 100+ have been randomized controlled trials and 95+ have been outcome studies. Notably, most of this research has been conducted in the past decade. The majority of these studies have concentrated on two acupoint tapping approaches: Emotional Freedom Techniques (EFT) and Thought Field Therapy (TFT). The focus of this paper is Clinical EFT.

Clinical EFT’s theoretical model is supported by a growing body of randomized controlled trials and systematic reviews. Meta-analyses report large effect sizes for PTSD (Cohen’s *d* ≈ 1.38–2.51 vs. passive controls) (Chen et al., 2025; Sebastian and Nelms, 2017; Stapleton et al., 2023). Systematic review and individual participant data meta-analysis indicated no significant difference between EMDR and other psychological treatments (of which EFT was included) in reducing PTSD symptom severity, achieving response, attaining remission, or reducing treatment dropout rate (Wright et al., 2024). For anxiety, systematic reviews indicate moderate-to-large pooled effects (Clond, 2016), with EFT performing similarly to CBT in direct comparisons (Jasubhai and Mukundan, 2018) including with high ability youth (Gaesser and Karan, 2017). Depression outcomes are consistent with these findings (Chatwin et al., 2016; Jasubhai and Mukundan, 2018), with meta-analyses reporting large effects (*d* ≈ 1.3–1.8) and sustained improvements beyond 90 days (Nelms and Castel, 2016). EFT has been shown to produce comparable reductions to CBT in anxiety and depression symptoms, as well as food cravings and dietary constraint for overweight and obese adults (Stapleton et al., 2016, 2017). In chronic pain, randomized trials show significant reductions in pain severity and interference, with benefits maintained at six-month follow-up and equivalent efficacy between in-person and online delivery formats (Salicru, 2025; Stapleton et al., 2022, 2025).

It is important to acknowledge that cognitive behavioral therapy (CBT) currently enjoys a far broader empirical foundation than EFT, with decades of research, hundreds of randomized controlled trials, and systematic reviews across multiple diagnostic categories (Cuijpers et al., 2021; Springer et al., 2018). By contrast, EFT’s evidence base, while growing rapidly, remains more recent and comparatively narrower, with fewer independent trials and smaller sample sizes. However, recent meta-analyses report large effect sizes for PTSD, moderate-to-large effects for anxiety and depression, and emerging evidence for chronic pain (previously discussed). In sum, the weight of evidence—especially where standardized Clinical EFT protocols were used—shows clinically meaningful and often large effects versus passive controls, and comparable outcomes versus active, established therapies in several head-to-head analyses. Continued improvements in trial rigor (blinding, preregistration, objective endpoints) will further consolidate these findings.

## The critiques of EFT

### The “purple hat” argument

Despite substantial evidence demonstrating the effectiveness of EFT, some critics have relied on the “purple hat argument” claiming that EFT in particular, works only due to nonspecific treatment effects (e.g., therapist intuition, Gaudiano, 2012) or the incorporation of techniques from other therapies (e.g., exposure; Boness et al., 2024). The “purple hat argument,” introduced by Rosen and Davison (2003) dismisses therapies that combine established evidence-based techniques with novel elements, suggesting that their effectiveness is often misattributed to these new components rather than established methods. Historically applied to criticize approaches like EMDR (Rosen et al., 2024) and EFT (Boness et al., 2024), these authors assume that such modalities derive their efficacy solely from cognitive-behavioral elements like exposure therapy.

Asserting that a novel therapy shares “active ingredients” with CBT offers little insight into the *quality, dosage, or synergistic interplay* of those ingredients. For example, while both EFT and Prolonged Exposure (PE) incorporate exposure-based techniques, their implementation differs substantially. EFT emphasizes *window of tolerance* management by addressing traumatic crescendos, whereas PE prioritizes extinction. Moreover, since EFT integrates additional components—such as mindfulness, self-compassion, and cognitive restructuring—in a unique, synergistic manner, it may constitute a distinct therapeutic modality rather than a derivative of CBT. Consequently, dismissing EFT as a “Purple Hat” therapy overlooks its potential as a comprehensive, evidence-based intervention addressing both psychological and physiological dimensions of distress. This raises questions about the field’s resistance to alternative paradigms. Rather than dismissing novel therapies *a priori*, critics might adopt a more inquisitively scientific stance toward their unique mechanisms and potential benefits. For instance, EFT’s integration of diverse psychological change agents warrants deeper investigation to elucidate its distinct contributions to therapeutic outcomes.

### The “pseudo-science” argument

Another persistent criticism of EP is that it should be considered pseudoscience. This stems from early explanations of its mechanisms, which were based on Eastern concepts of energy flows and the stimulation of acupuncture meridians —structures that had not been anatomically identified in the body at that time.

This critique persists today despite emerging anatomical research on meridians (e.g., Li et al., 2021; Stefanov et al., 2013), and it is sometimes accompanied by hyperbolic claims that clinicians using these approaches are unethical (Boness et al., 2024). However, these critiques have been disputed and debunked (Feinstein, 2019, 2021, 2024). Additionally, proponents of EFT have responded in two ways. First, by highlighting dismantling studies that show the actual tapping on acupoints as an active ingredient (Feinstein, 2023, 2024). Second, by presenting research supporting various physiological and neurological mechanisms (Feinstein, 2023, 2024, 2025b).

The discourse surrounding the controversies and disagreements about EFT often centers disproportionately on the act of “tapping.” Critics, such as Boness et al. (2024), appear to interpret EFT as a simplistic process where clients merely tap on acupuncture points while focusing on a problem, resulting in incredible outcomes. This reductive view understandably invites skepticism. Compounding this issue, popular descriptions of EFT on websites and platforms like YouTube frequently reinforce this oversimplified narrative, further detracting from a nuanced understanding of the method (see discussion in the Active Ingredients section for several of these nuances).

Even within academic circles, serious scholars inadvertently perpetuate the notion that EFT’s efficacy is primarily attributable to “meridian tapping”. While discussions emphasizing meridian tapping (Feinstein, 2024) have merit, such a singular focus neglects the broader clinical and psychological dimensions integral to EFT’s therapeutic application. Experienced EFT practitioners recognize that tapping itself becomes routine once mastered, and is only one aspect of treatment. Core clinical skills—deeply registering a client’s self-understanding and internal experience, identifying appropriate targets for intervention, and tailoring treatment strategies to individual needs—are critical components of EFT. In this sense, EFT can and should be conceptualized as a form of psychological intervention or therapy.

With the exception of exposure and memory reconsolidation (Church et al., 2022; Feinstein, 2021, 2025b; Stapleton et al., 2020), the literature offers limited attention to important psychological active ingredients or mechanisms that substantially contribute to EFT’s effectiveness. This paper seeks to address this gap by examining the psychological processes necessary for achieving clinical outcomes with EFT and the specific interventions embedded within its framework. While we concur that EFT incorporates techniques common to other evidence-based therapeutic approaches (e.g., exposure therapy), we argue that this integration represents a strength rather than a flaw. Clinical EFT synthesizes psychologically active components from various modalities in a unique manner, creating a synergistic combination of active ingredients.

### The “it is not psychological therapy” claim

With the “lack of scientific evidence,” the “pseudo-science,” and “purple hat” arguments debunked, a new tactic has emerged. The decades’ old definition of a psychological interventions was recently revised (Tolin et al., 2024) as part of a gate-keeping strategy on the part of APA’s Division 12. This had the desired effect of disqualifying Clinical EFT, as well as any other somatic approaches from being considered for analysis as evidenced-based. We agree with Feinstein (2025c) that this strategy has serious flaws and many previously accepted evidenced-based approaches such as mindfulness-based interventions and EMDR, may no longer be considered for evaluation for the Tolin et al. 2015) criteria for an evidenced based psychological therapy (see https://societyofclinicalpsychology.org/resources/psychological-treatments/). Indeed, at the time of this paper only five therapies (CBT for gambling, substance use and insomnia, contingency management for substances and exposure/response prevention for obsessive compulsive disorder) met the new revised criteria.

This paper advocates for a more comprehensive understanding of EFT as a psychological intervention with somatic components that transcends reductive interpretations focused solely on tapping. By mapping Clinical EFT’s active ingredients to the psychological and physiological mechanisms that may account for these observed outcomes. We aim to clarify how its integrative structure produces therapeutic change. By emphasizing the interplay of clinical acumen, psychological strategies, and somatic interventions within EFT’s framework, we aim to illuminate its multifaceted nature and provide a robust foundation for future research and application.

## Active ingredients in emotional freedom techniques (EFT)

The active ingredients of a therapy are the specific interventions and components included in the treatment to maximize outcomes. We suggest that in Clinical EFT these elements work synergistically to produce therapeutic effects. Clinical EFT incorporates multiple active ingredients that work in combination to produce therapeutic change. Table 1 provides an overview of these components. In the following section of this paper, we will describe how these active ingredients are linked to four mechanisms of change.

**Table 1:** Proposed active ingredients of clinical EFT.

Active ingredient	Brief description
1. Mindfulness, self-compassion and externalization (distinguishing self from the problem)	“Even though X, I accept myself” phrasing
2. Systematic exposure within window of tolerance	Titrated recall and processing of traumatic memory broken into crescendos
3. Somatic intervention (acupoint tapping)	Tapping on meridian (acupuncture) points to modulate arousal
4. Leveraging demand characteristics, hope and positive expectancy	Cycling of distress rating and tapping sequences
5. Shifting/titrating awareness	Strategic questioning, selection of experiential targets for processing, and technique selection
6. Utilization approach	Therapist utilizes the client’s words, emotions and sensory experience to guide rounds
7. In-session processing	Emphasis on real-time regulation vs. versus exclusive reliance on cognitive restructuring and prescribed behavioral change procedures

### Mindfulness, self-compassion and externalization

The therapeutic frame of EFT includes 3 active ingredients: *creating a mindful orientation, emphasizing self-compassion, and externalizing the problem from one’s sense of self*. These foundational elements are used particularly during the “setup” phase of the basic protocol (although they occur throughout treatment). During this phase, a target issue (e.g., symptom, emotion, or memory) is identified, including various “aspects” (e.g., sensations, cues, self-talk, emotions). The client repeats a setup phrase such as, “Even though I [description of target], I deeply and completely accept myself,” while tapping on the side of the hand. This process fosters:

- Mindfulness: encouraging a meta-awareness of one’s experience.
- Self-compassion: promoting acceptance and kindness toward oneself.
- Externalization: separating the problem from the self, aligning with Narrative Therapy principles

This one procedure, that is repeated many times throughout treatment, interweaves all three of these active ingredients. Externalizing the problem is an approach used in Narrative Therapy (White and Epston, 1990) that focuses on separating the identity of the person from the problem. Similar principles occur within Acceptance and Commitment Therapy (Hayes et al., 2012). The phrase “Even though *x*, *y* and *z* experiences are happening, I accept myself” builds the idea that “I” am not the problems of *x*, *y* and *z*. This also creates a mindful position of being separate from and meta to the various experiences happening. The last part of the phrase “accept myself” is building self-compassion (Germer and Neff, 2015). Building a mindful, self-compassionate awareness where the client’s sense of self is more than their aversive experience is part of the frame of all of EFT’s different techniques.

### Exposure within window of tolerance

Trauma Informed approaches have come to recognize that exposure while keeping intensity within the therapeutic window of tolerance (Briere and Scott, 2006, 2014; Siegel, 1999a, 1999b) is crucial if the goal is to achieve reduction of symptoms. The challenge clinically is to titrate the exposure to the uncomfortable emotions and experiences so as not to overwhelm the client. If the therapy is too “cold” there is too much avoidance and nothing may happen. If the therapy is too “hot,” the client becomes overwhelmed and risks shut down. Clinical EFT employs a unique approach to exposure that combines these two active ingredients. Clinical EFT creates exposure withing the window of tolerance by deconstructing memories into emotional crescendos and addressing each aspect while maintaining the client within their window of tolerance.

In the *Tell the Story* technique, when a client recalls a traumatic memory, the practitioner structures the telling of the story into its component parts, focusing on *specific elements of the memory one at a time*. Each component part is organized around a crescendo of intensity (Briere and Scott, 2014). A key instruction is to ask the client to cease telling the story as soon as they become aware of any distress – the client is also advised that the therapist or practitioner will stop them if they notice signs of distress. The client then taps on acupressure points while recounting each fragment of the memory, strategically targeting their physiological and emotional response to each part of the traumatic experience. The goal is to reduce the SUD (Subjective Units of Distress) to zero (or near zero) for each crescendo of the memory before moving on to the next part of the memory. This approach is well-supported in the literature under the concept of “titrated exposure” (e.g., Briere and Scott, 2014; Gerbode, 2006).

By slow and methodically tapping and reducing the SUD to zero for each fragment of the memory, the client is prevented from becoming overwhelmed. The physiological mechanisms by which the somatic activation of tapping works has been delineated by Feinstein (2025a, 2025b). The dysregulating effect of the memory begins to decrease as each crescendo is reduced to a SUD of zero or near zero. Furthermore, the client gains confidence in the ability of EFT to regulate the distress. By tapping on each component of the memory until the distress is eliminated, EFT achieves what Acceptance and Commitment Therapy (ACT) aims for: reducing the tendency to avoid difficult emotions and encouraging a direct, mindful engagement with one’s difficult emotional experiences (Hayes et al., 2012). This tendency, termed emotional avoidance, allows for complete processing of the entire traumatic memory. This ability of EFT to reduce emotional avoidance is a proposed mechanism of change and is discussed soon.

Therapists too, are also expected to deal with emotional avoidance and stay within their own window of tolerance. Because the therapist is almost always tapping on themselves while guiding the client, they maintain rapport and stay regulated. The calmness of the therapist may then assist to co-regulate the client. The importance of interpersonal coregulation has been discussed by Porges (2021, 2022) via the vagal system and others via mirror neurons (Patel, 2024). Therapists who become dysregulated may inadvertently trigger the client to engage in emotional avoidance to protect themselves or the therapist. Schore’s (2012, 2022) extensive research on interpersonal neurobiology provides another foundation for understanding the need for this coregulation. When therapists use self-regulation techniques such as tapping, their regulated nervous system becomes a template that clients unconsciously attune to through mirror neuron activation and autonomic synchronization. This creates “embodied coregulation”—an active therapeutic presence that sends safety cues to the client’s system, helping shift them from dysregulated states toward social engagement. The process is bidirectional, protecting the therapist from becoming dysregulated while providing consistent coregulation that, over time, becomes internalized by the client through neuroplastic changes, building their own self-regulation capacities.

### The use of somatic activation to reduce emotional dysregulation

Most experts in trauma treatment have come to recognize the importance of engaging the body in some form as part of treatment (Kezelman and Stavropoulos, 2019; Kuhfuß et al., 2021; Ogden and Fisher, 2015; Schwartz, 2021; van der Kolk, 1994, 2014). Feinstein (2019, 2025c) reviewed multiple studies indicating that acupoint stimulation in EFT leads to rapid reductions in limbic system arousal, as measured by fMRI and other brain imaging techniques. The published research (see Church et al., 2022 for a summary) suggests significant reductions in biochemistry markers after EFT, indicating a shift toward parasympathetic dominance.

Research suggests that autonomic nervous system (ANS) dysregulation, rather than the traumatic event itself, is a stronger predictor of later psychological symptoms (Jokić et al., 2023; Kolacz et al., 2020a, 2020b). Schwarz (2018) proposed a link between meridian point tapping and increased heart rate variability (HRV), indicative of enhanced ventral vagal regulation. The author notes that the phenomenological responses to tapping suggest improvements in ventral vagal activity, which plays a critical role in signaling safety to the brain. Clients completing Clinical EFT often express pleasant surprise and puzzlement when revisiting previously distressing events. A common phrase that client’s use is “where did it go?” When this question is unpacked, “it” invariably refers to the client’s bodily sense of associated danger signals and corresponding symptomology. This shift suggests an impact on perceived ANS safety. van der Kolk (2014) emphasizes the necessity for complex trauma survivors to restore a positive relationship with their bodies. Beyond any meridian system benefits, the act of tapping and experiencing a positive bodily response contributes to regaining a sense of bodily agency and well-being. That said, the type of body activation may, in the future, prove less critical than the engagement of the body itself.

Clinical EFT, involving meridian point tapping and engagement of internal senses, has demonstrated that tapping on meridians serves as an active ingredient in therapeutic change (Church et al., 2018a, 2018b). The physiological mechanism of this appear to include mechanosenosory transduction and neural mechanisms (Feinstein, 2025a, 2025b, 2025c; Salicru, 2025) but are beyond the scope of this paper. Other body-oriented therapies utilize different activation methods—EMDR employs bilateral stimulation through eye movements or alternating tactile stimulation, Brainspotting (D’Antoni et al., 2022; Talbot et al., 2023) uses specific eye positions to access trauma-related brain activation, and Havening (Sumich et al., 2022) incorporates eye movements, gentle touch combined with visualization. While EFT’s meridian-based tapping approach may offer a distinct somatic pathway, it may be that all somatic “roads lead to Rome” (i.e., regulation). The relative importance of different somatic activation techniques is an area for future research.

### Leveraging demand characteristics and positive expectancy

Martin Orne’s concept of *demand characteristics* (1962) highlights how subtle cues in therapy influence clients’ behavior and expectations. Traditionally seen as a bias to overcome, these cues can instead be leveraged to promote healing. Therapists can intentionally foster positive expectancy by conveying belief in clients’ capacity to improve, reinforcing hope and engagement (Frank, 1973). Research on the placebo effect and common factors in psychotherapy demonstrates that belief in the efficacy of treatment significantly enhances outcomes, with client expectancy accounting for approximately 15% of therapeutic improvement (Cuijpers et al., 2019; Lambert, 1992). By strategically maximizing demand characteristics, therapists create an environment where recovery feels possible, transforming a potential limitation into a tool for facilitating meaningful therapeutic change. One way EFT leverages demand characteristics and positive expectancy is through use of a TOTE structure (Test–Operate–Test–Exit; Miller et al., 1960). EMDR employs a similar iterative structure, although neither EFT nor EMDR explicitly referenced the TOTE model in their development; rather, both appear to have converged on its functional utility. Originally formulated as a cognitive framework for problem-solving and goal-directed behavior, the TOTE model provides a useful heuristic for understanding how psychotherapies organize experiential change. Any therapeutic approach that asks clients to rate subjective distress and then applies an intervention until distress is reduced to zero or near zero is, in effect, operating within a TOTE framework.

In the *Test* phase, the client’s current emotional or physiological state is assessed relative to a desired state. In EFT’s *Tell the Story* technique, this corresponds to identifying and rating distress (SUD) associated with specific crescendos of a traumatic memory. If distress remains above zero, the *Operate* phase is initiated, during which targeted interventions are applied. In EFT, this involves identifying specific contributing aspects of experience—such as images, sounds, bodily sensations, emotions, and internal dialogue—and guiding the client through acupoint tapping while attending to these elements. This deconstruction of experience is highly individualized and requires considerable clinical skill, underscoring the inadequacy of reductive characterizations of EFT as “just tapping.”

Following each intervention, a second *Test* occurs as the client re-rates distress. If SUD remains elevated, the cycle returns to Operate until distress is substantially reduced. The *Exit* phase is reached when the client can recall the memory with minimal or no distress, indicating effective regulation and processing. The consistent use of SUD ratings, with Exit defined by low or absent distress, reinforces expectancy and the perception that resolution is achievable. It is likely not coincidental that both EMDR and acupoint tapping protocols that combine somatic intervention with a structured TOTE process—and are among the most effective and cost-effective trauma treatments (Mavranezouli et al., 2020a,b).

### Shifting and titrating the focus of awareness

The next active ingredient is titrating the focus of awareness either through the selection of specific EFT techniques or the tactical and strategic use of questions. The exploration of the “aspects’ of a memory or an experience is considered to be one of the top five EFT techniques by practitioners (Church et al., 2022). This process involves deconstructing the distinct emotional cognitive, sensory, and physiological components that together constitute a client’s experience. The client is asked to focus on those component parts while tapping. Furthermore, this approach throughout the rounds of tapping facilitates clients focusing awareness on and often attaching verbal descriptions to the different aspects of their unfolding experiences within an emotional context of relative calm and acceptance. In many cases, this may be the first time that those experiences have had verbal descriptions attached to them, allowing a new level of processing to occur (Lane et al., 2015).

The Tell the Story technique described above titrates awareness very differently than a typical recounting of a traumatic event to prevent overwhelm. If even more protection from overwhelm is deemed necessary, then the “Tearless Trauma Technique” is utilized before Tell the Story technique (Stapleton and Janzen, 2025) which allows clients to work on traumatic events without fully diving into the emotional intensity that the details of such memories might evoke. By using distancing metaphors and tapping on the *perceived* intensity of the memory, clients can gradually reduce the emotional charge associated with the trauma, ensuring they remain within their window of tolerance. Therapist questions are not simply passive requests for information; they actively determine what a client notices, how they interpret their experience, and where they direct their energy. Both cognitive psychology and Ericksonian hypnosis highlight the power of questions—cognitive psychology emphasizes their influence on attention and memory, while Ericksonian hypnosis focuses on engaging the subconscious and reducing resistance to change.

Awareness is inherently selective, and questions serve as tools to titrate this focus by:

1. Narrow awareness (focus on a specific thought or sensation),
2. Broaden awareness (explore connections or new perspectives), and
3. Shape awareness (frame focus to align with therapeutic goals).

Well-trained clinical EFT practitioners use these strategies to titrate intensity and maintain regulation throughout treatment.

### Utilization as an active therapeutic ingredient

Utilization refers to the deliberate therapeutic practice of accepting and strategically incorporating whatever the client presents—symptoms, resistance, metaphors, affective responses, language patterns, or somatic reactions—into the intervention itself (Erickson, 1959). Within the proposed framework of Clinical EFT (Table 1), utilization functions as a coordinating active ingredient that dynamically integrates and optimizes the operation of other therapeutic components. Rather than opposing or correcting client material, the therapist treats it as diagnostically and therapeutically informative. This non-oppositional stance reduces emotional avoidance, supports mindfulness and self-compassion by validating present-moment experience, and preserves engagement within the window of tolerance by preventing escalation of threat responses.

Importantly, utilization enhances therapist–client attunement by requiring continuous, moment-to-moment tracking of the client’s emotional and physiological state, a process shown to be central to affect regulation and therapeutic change (Schore, 2012). While utilization shares surface similarities with common-factor constructs such as unconditional positive regard (Rogers, 1957), its clinical function extends beyond alliance-building to actively shaping the form, timing, and focus of therapeutic interventions.

From a mechanistic perspective, utilization enhances the effectiveness of other active ingredients by guiding exposure dosage, directing shifts in awareness, and informing in-session processing in real time. By continuously incorporating client feedback, utilization increases salience for implicit emotional learning systems while minimizing counteractive learning. When feared, avoided, or self-protective responses are met with acceptance and incorporated as resources, a subtle but meaningful prediction error emerges—safety, validation, or agency is experienced where threat or invalidation was anticipated. This discrepancy aligns with experimentally identified conditions required for destabilizing emotional learnings prior to memory reconsolidation (Lane et al., 2015; Ecker and Vaz, 2022).

Attuned therapeutic responsiveness further amplifies this effect by supporting right-brain-to-right-brain communication and autonomic coregulation, thereby maintaining access to emotionally salient material without triggering defensive shutdown (Schore, 2012). In this way, utilization functions as a process-level catalyst that amplifies demand characteristics and positive expectancy while supporting physiological regulation.

In Clinical EFT, utilization is operationalized through specific, observable practices that directly influence therapeutic pacing, attunement, and precision. These include the disciplined use of the client’s exact words in setup phrases, the prioritization of emerging aspects over rigid adherence to predetermined targets, and the continuous use of SUD fluctuations to titrate exposure and guide intervention timing. Emotional reactions—such as spikes in distress, unexpected affective shifts, or somatic responses—are treated as clinically meaningful instructions rather than errors or deviations from protocol.

While striving for the SUD to eventually reach zero, it is crucial to understand that practitioners do not expect or need the SUD to go to zero immediately. Practitioners expect that it will require multiple rounds of the TOTE process over each crescendo to deconstruct the different aspects of the traumatic experience. The therapist accepts and utilizes any client feedback as necessary and useful information to guide the next tapping round. Attending to and utilizing the different components aspects of the memory is a significant part of clinical presence and acumen. Multiple rounds of tapping on the aspects of each crescendo of a traumatic memory is common in order deconstruct the experience so that the intensity of each crescendo reaches zero.

This utilization-oriented stance allows EFT practitioners to remain finely attuned to the client’s nervous system state while sustaining therapeutic momentum, particularly in trauma-focused work where mis-attunement can precipitate overwhelm or disengagement. Viewed in this way, Erickson’s utilization is not ancillary to EFT technique but constitutes a core process variable that directly shapes attunement, regulation, expectancy, exposure tolerance, and the conditions under which memory reconsolidation and durable therapeutic change are most likely to occur.

### In-session experience (rather than reliance on homework)

CBT and many other evidence-based therapies rely heavily on homework assignments to generalize skills, test hypotheses, promote independence, and consolidate treatment gains. Meta-analytic findings indicate a significant relationship between homework compliance and outcomes, with one review showing that clients who completed homework achieved better results than approximately 70% of those who did not (Prasko et al., 2022). Despite this, homework non-compliance remains a persistent clinical problem, with adherence rates commonly ranging from 20 to 50% among adult clients. Indeed, failure to complete homework is frequently cited as a major contributor to suboptimal outcomes and treatment dropout in CBT (Tang and Kreindler, 2017).

In contrast, Clinical EFT (like EMDR) places primary emphasis on *in-session experiential processing*, representing a meaningful shift in therapeutic strategy. Rather than relying on repeated between-session practice to gradually counteract symptoms, EFT incorporates active regulation, exposure, and cognitive-emotional processing directly within the session itself. This approach allows traumatic memories, emotional learnings, and maladaptive schemas to be activated and transformed in real time, reducing dependence on client adherence outside the therapy hour.

This distinction can be understood through the framework articulated by Ecker and Vaz (2022), who differentiate *incremental change* from *transformational change*. Incremental change—often achieved through extinction, coping strategies, or counteractive learning—typically results in partial symptom reduction and requires ongoing effort to maintain gains. Transformational change, by contrast, occurs through memory reconsolidation (discussed later under mechanisms); when this process is completed, the original emotional learning no longer generates symptoms, distress resolves durably, and continued effort is no longer required to sustain improvement. When Clinical EFT is used to process traumatic memories and core schemas, clinical outcomes frequently resemble the pattern of transformational change described by Ecker and Vaz (2022). EFT’s active ingredients are designed to produce immediate physiological regulation and emotionally corrective experiences during the session itself, facilitating reconsolidation rather than incremental symptom management. As a result, therapeutic gains are more likely to be consolidated within the session, reducing the need for extensive homework. Given the well-documented challenges of homework non-compliance in approaches such as CBT, the in-session focus of EFT and EMDR may offer meaningful advantages for treatment adherence, efficiency, and long-term outcomes.

## Mechanisms of change (the intrapersonal processes that must change to achieve a clinical response)

Whereas the active ingredients of a therapy are the specific interventions and other components included in the treatment (e.g., positive expectations, therapeutic alliance, therapist caring), the mechanisms of change refer to the underlying processes through which a treatment produces improvement—processes within the client, not techniques applied by the therapist. The active ingredients of EFT (beyond non-specific factors shared by all effective therapies) activate four primary mechanisms of change: reducing emotional dysregulation, reducing experiential avoidance, facilitating cognitive restructuring, and enabling reconsolidation. These four mechanisms, in turn, serve as pathways to clinical benefits such as reduced depression, anxiety, PTSD symptoms, and addictive behaviors. These mechanisms are not isolated effects but emergent processes arising from the interaction of multiple active ingredients. Each mechanism can be linked to specific therapeutic outcomes, as well as to clusters of active ingredients described earlier in this paper. The mechanisms proposed below are grounded in contemporary trauma and psychotherapy research and reflect EFT’s distinctive integration of psychological, somatic, and neurophysiological processes.

### Reduction of dysregulation

**Figure 1:**
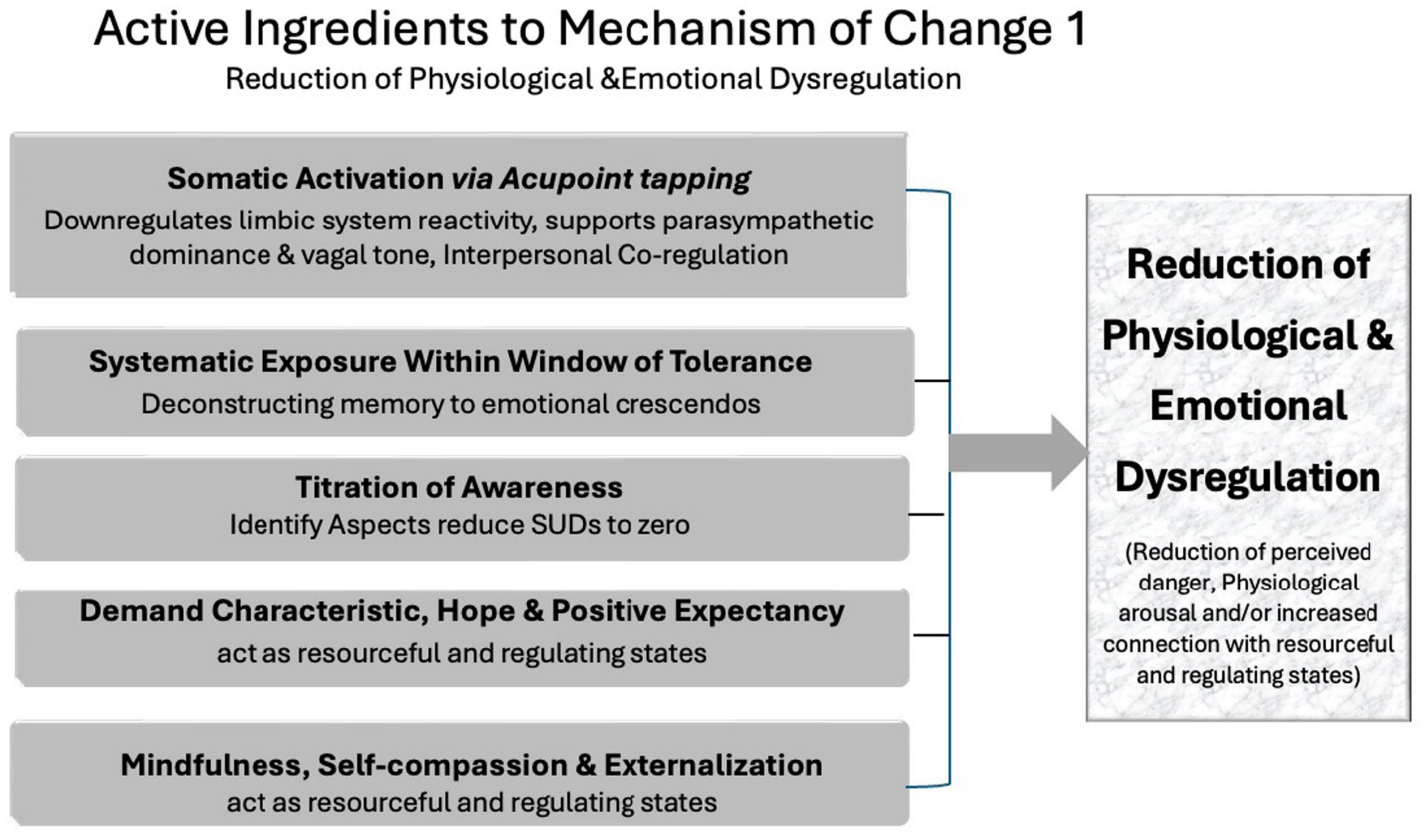
EFT mechanism of change 1: reduction of physiogical and emotional dysregulation.

The first mechanism is the reduction of emotional and physiological dysregulation. Dysregulation occurs when clients become overwhelmed by fear, anxiety, or other affective states, typically due to impaired autonomic nervous system (ANS) function or unresolved trauma. EFT addresses dysregulation through a synergy of active ingredients, as summarized in the middle and right columns of Figure 1. These include:

- *Somatic activation via acupoint stimulation* which rapidly downregulates limbic system reactivity, and supports parasympathetic dominance and vagal tone (Church et al., 2022; Feinstein, 2019; Schwarz, 2018).
- *Interpersonal co-regulation* supported by therapists tapping alongside clients and maintaining calm affective presence (Porges, 2021).
- *Positive expectancy and hope* (Frank, 1973; Miller et al., 1960; Orne, 1962) that act as resourceful and regulating states.
- *Titration of awareness*, via techniques such as Tell the Story and Tearless Trauma, which help maintain clients within their window of tolerance.
- *Self-compassion, mindful acceptance and unconditional positive regard* are resourceful states that decrease shame and fear-based reactivity.

Together, these processes signal safety to the brain and body, creating conditions for regulated states to emerge. As a result, clients experience fewer intrusive symptoms and gain greater emotional flexibility, which lays the foundation for deeper therapeutic work.

### Reduction of emotional avoidance

**Figure 2:**
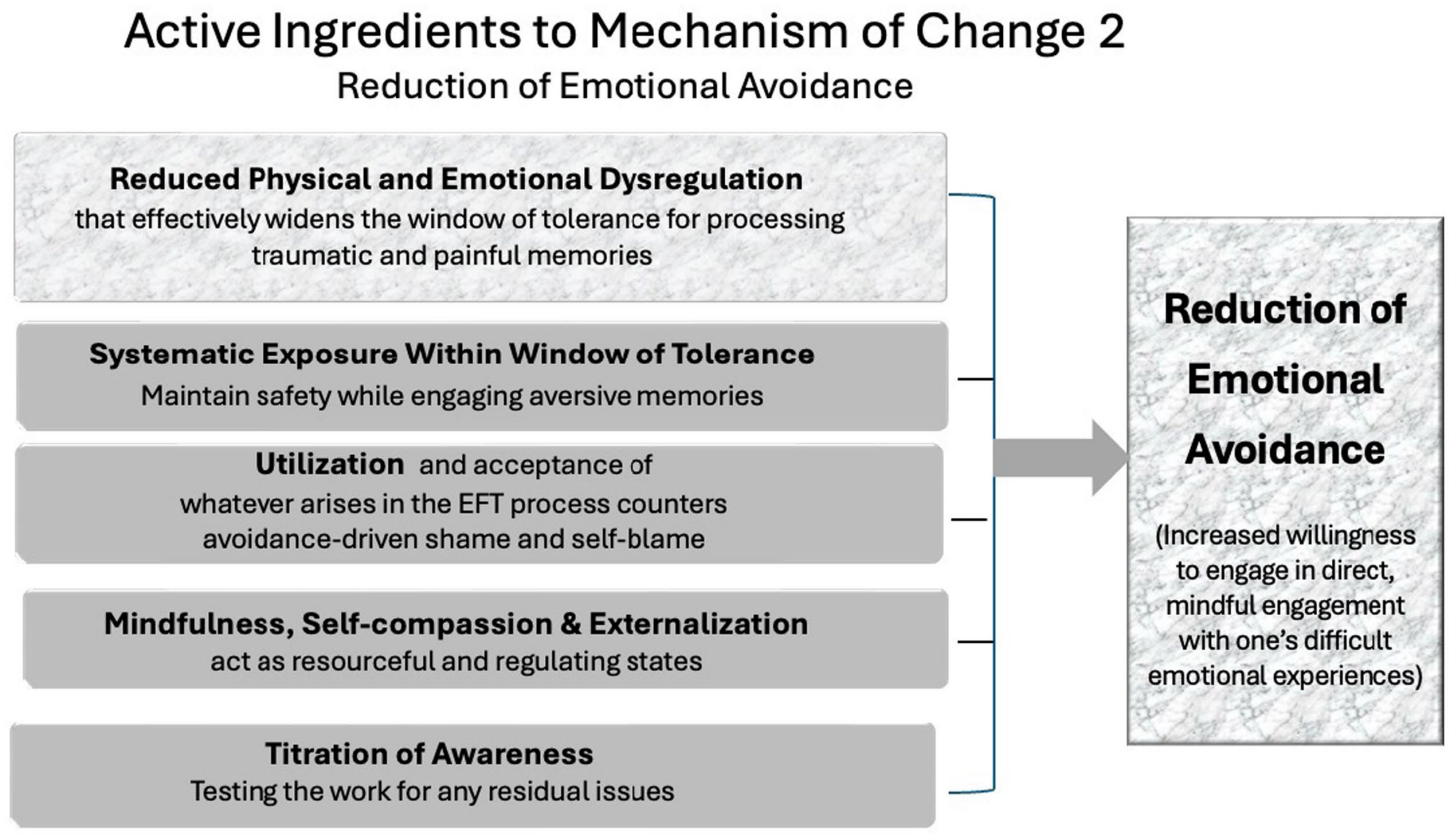
EFT mechanism of change 2: reduction of emotional avoidance.

The second mechanism involves a systematic reduction of emotional avoidance (Hayes et al., 2012). Many trauma survivors cope by avoiding painful internal states, memories, or relational cues. However, sustained avoidance perpetuates symptomatology and prevents processing of traumatic material. EFT intervenes here by through a unique approach to exposure that is deeply embedded within its procedural structure (see Figure 2) and includes:

- Reduced dysregulation that effectively widens the window of tolerance for traumatic and painful memories.
- Graduated exposure within the client’s window of tolerance
- Emotional validation and containment, which encourages the client to engage painful content with support.
- The therapeutic stance of acceptance and utilization of whatever arises in the EFT process which counters avoidance-driven shame and self-blame.
- Structured, titrated storytelling techniques that maintain safety while engaging aversive memories.
- Once the memory has been treated the client and therapist “test the work” by reviewing the memory again to test if any part of the memory can still create discomfort^1^. Any response is treated as a positive outcome. If the SUD remains at zero then this is further confirmation that the work is complete (and enhances reconsolidation). If the SUD go up during any part of the recall, it’s just an opportunity to clean out anything that was missed or avoided. A little more EFT is done until the final test leads to no discomfort during any part of the memory.

By breaking traumatic memories into manageable segments and eliminating distress at each step, EFT enables clients to face what was previously impossible. This process increases emotional resilience and self-efficacy, while also facilitating greater trust in the therapeutic process.

### Cognitive and emotional restructuring

The third mechanism is the restructuring of cognitive and emotional associations. This process involves the development of new meanings, emotional responses, and narratives around previously distressing cues. Drawing on both cognitive-behavioral, somatic and hypnotic traditions, EFT fosters restructuring through:

- Experiencing emotional regulation and safety in the presence of previously aversive stimuli.
- New associations between previously aversive stimuli and safety-based somatic experiences (e.g., tapping while recalling trauma).
- Reframing and externalization, integrated into the setup phrase and cognitive interweaves.
- Increased access to adaptive self-talk and inner dialogue, supported by mindful awareness and self-compassion.
- Shifting focus of awareness to component parts of aversive experience and strategic questioning, which reshapes perception and attribution patterns.
- New recall of lost events or context from traumatic or painful memories that changes meaning.

**Figure 3:**
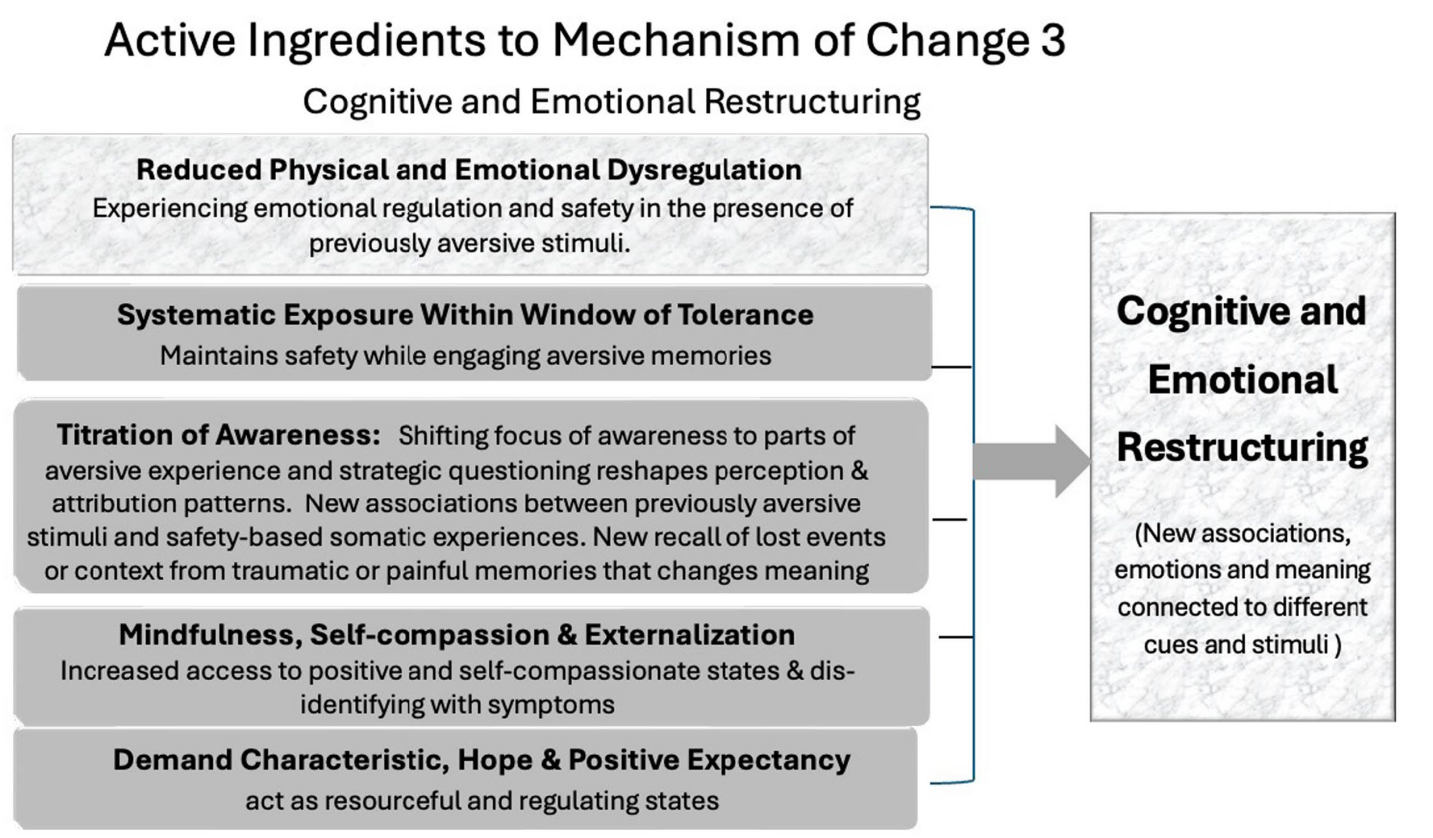
Mechanism of change 3: cognitive and emotional restructuring.

Through this process, clients come to reinterpret their experiences, shift blame away from the self, and develop more flexible, empowering schemas. These new patterns are emotionally encoded, increasing their staying power beyond the intellectual level (see Figure 3).

### (Memory) reconsolidation

**Figure 4:**
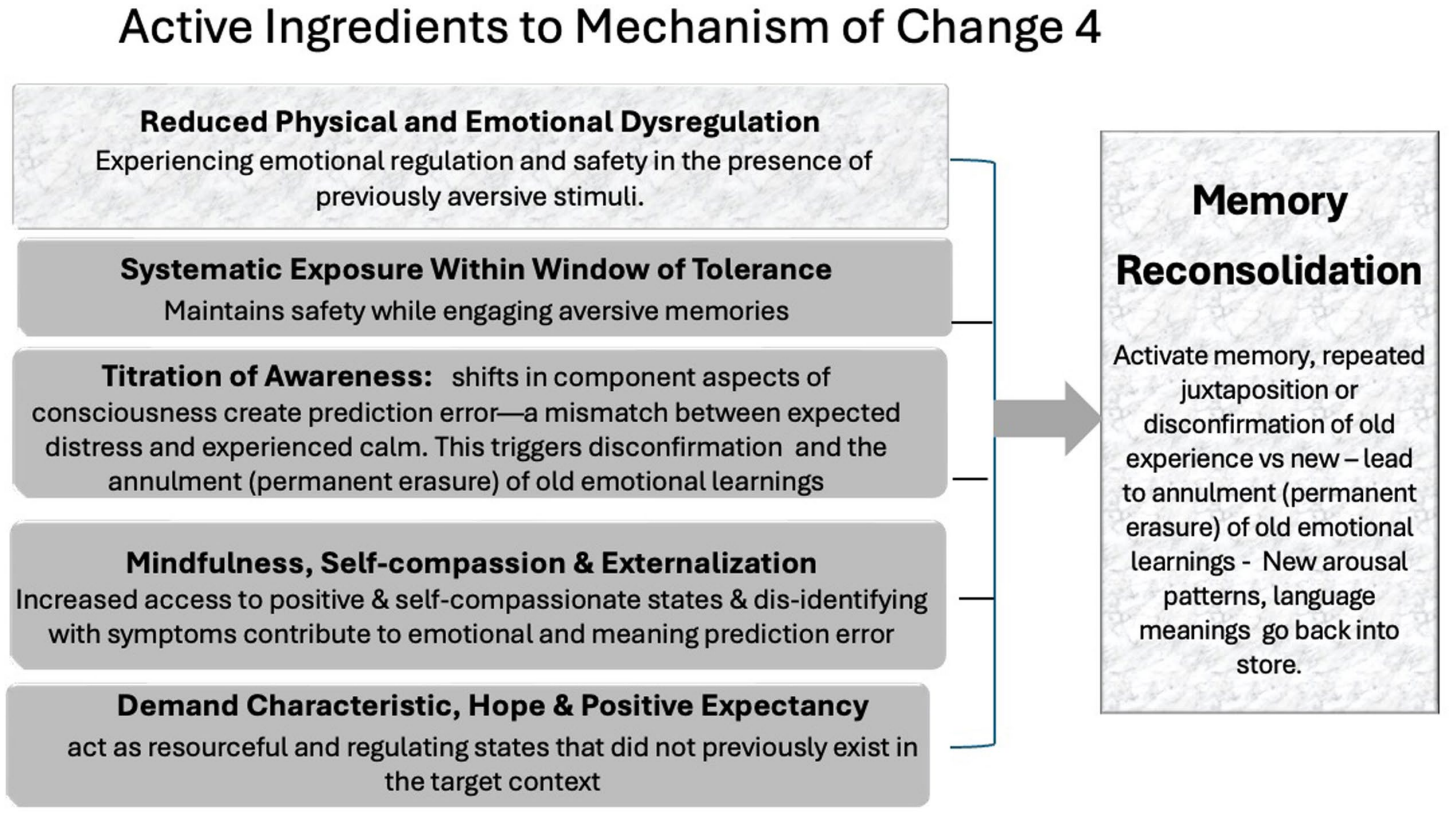
Mechanism of change 4: memory reconsolidation.

The final mechanism is *memory reconsolidation*. In EFT, the mechanism of *memory reconsolidation* is engaged through a sequence of active ingredients that enable the *permanent erasure*—or *annulment*—of maladaptive emotional learnings (Ecker, 2018; Ecker and Vaz, 2022). The process begins with the *reactivation* of a target memory through titrated exposure, followed by a mismatch experience: the client systematically recalls distressing material within a precise therapeutic structure while unexpectedly feeling calm, safe, or self-compassionate due to increased physical and emotional regulation, increased access resourceful states of mind and disidentifying with the problem. This mismatch violates the brain’s expectation and triggers a prediction error, destabilizing the original memory. When this contradiction is repeated within the session, the brain rewrites the emotional learning, resulting in *annulment* and permanent erasure at the neural level. We acknowledge that schemas and mental models may be reconsolidated as well as memories (see Lane et al., 2015) and use the term *reconsolidation* to reflect all of these. There are numerous methods to achieve the process. But “what matters to the subject’s brain is not the concrete procedure …, but rather the subjective experience created by the procedure” (Ecker, 2018, p. 16) (see Figure 4).

Ecker and Vaz (2022) distinguish incremental change from transformational change. Incremental change—common in many therapies and often achieved through extinction, coping skills, or counteractive strategies—produces partial symptom reduction. Such change usually requires ongoing efforts to maintain the therapeutic gains. In contrast, transformational change occurs through reconsolidation. When reconsolidation is completed, the original emotional learning no longer generates symptoms, distress disappears abruptly and durably, and no ongoing effort is required to maintain gains. When Clinical EFT is utilized to process traumatic memories and schema, the results usually parallel the descriptions of transformational change.

### Implicit to explicit memory integration

Contemporary affective neuroscience distinguishes among multiple interacting memory systems relevant to this process, including implicit memory, explicit memory, autobiographical (episodic) memory, and semantic memory (Lane et al., 2015). EFT appears to engage and integrate these systems in a way that allows maladaptive emotional learnings to be fundamentally transformed rather than merely suppressed or cognitively overridden.

In EFT, the target memory is accessed and activated through techniques such as *Tell the Story* that reliably reactivate emotionally encoded autobiographical memories. Reconsolidation is achieved through the convergence of the three preceding mechanisms:

- The client is kept in a regulated and safe state through tapping, co-regulation, and titrated exposure.
- Through the various active ingredients discussed earlier, emotional avoidance is reduced and clients are encouraged to have direct, mindful engagement with their emotional experiences.
- Restructuring of emotional and cognitive experiences—such as feeling calm, safe, or empowered while recalling previously distressing material—are introduced, juxtaposing and contradicting the original emotional learning. The procedure is repeated until distress is eliminated, increasing the likelihood that the new learning is durably encoded.

Many traumatic experiences are initially encoded primarily in *implicit memory systems*, characterized by intense affective and physiological responses with little narrative structure or verbal representation. Such memories may be accompanied by fragmented images, bodily sensations, or global emotional meanings (e.g., terror, shame), often coupled with rigid or highly negative verbal descriptions when language is present at all.

The slow, titrated review of details in the Tell the Story technique—combined with increasing physiological regulation—allows these implicit emotional memories to become progressively more explicit—In other words, the focus of awareness begins to shift. As the client revisits the experience in small segments while remaining within the window of tolerance, sensory, emotional, and physiological elements are integrated with verbal labeling, temporal sequencing. Importantly, this making explicit does not occur in a neutral state but in the presence of new, resourceful emotional conditions, particularly safety, calm, and self-compassion. All of these changes become reconsolidated when the memory goes back into store.

Because emotional memories are stored as integrated networks involving affect, physiology, and meaning, reconsolidation in EFT does not merely alter the cognitive narrative of the memory (although that often happens spontaneously). The emotional and physiological components—including autonomic arousal patterns—are also updated. The reconsolidated memory thus contains not only revised interpretations but also new embodied responses, such that recalling the event no longer triggers the original fear-based or shame-based state.

### Autobiographical and semantic memory: tabletops and table legs

Lane et al. (2015) describe autobiographical memory as explicit, episodic memory tied to personally experienced events, and semantic memory as generalized knowledge, beliefs, and meanings abstracted from repeated experiences. EFT’s distinction between “table legs” (specific events) and “tabletops” (global beliefs or schema) closely parallels this neuroscientific distinction.

In EFT, identifying table legs is functionally equivalent to making explicit the autobiographical memories that support a particular semantic belief structure. For example, a belief such as *“I’m unlovable”* represents a semantic memory—an abstracted meaning derived from multiple emotionally charged experiences. EFT systematically traces this semantic structure back to the specific autobiographical memories that gave rise to it.

### Systemic deconstruction of semantic beliefs

The EFT process of deconstructing a global belief into its supporting memories involves applying Tell the Story or related techniques to each relevant traumatic autobiographical memory. As each memory is activated under conditions of safety and regulation, it becomes labile and available for reconsolidation. New emotional experiences—such as self-acceptance, compassion, or adult perspective—contradict the original learning encoded in that memory.

Crucially, this process operates at two interconnected levels of reconsolidation:

1. *Autobiographical reconsolidation*: Each individual traumatic memory is updated so that it no longer carries the same emotional charge or physiological threat response.
2. *Semantic reconsolidation*: As the autobiographical “legs” are transformed, the overarching semantic belief loses its evidentiary support. The belief itself—*“I’m unlovable”*—is reconsolidated into a more adaptive, flexible, and meaning, “*I am lovable*.”

Thus, EFT does not merely challenge maladaptive beliefs through cognitive disputation. Instead, it *alters the emotional memory network from which those beliefs originally emerged*.

Whenever the new reconsolidated memory is activated (consciously or unconsciously) it is now encoded *without* the cues and meaning of danger and with the new cues of safety, new affective states, new verbal descriptions and meanings. Not only is the autobiographical memory permanently change, so is the semantic meaning of that memory. The new semantic meaning then increase the likelihood of more resourceful perception of the current situation the client find’s themselves in, along with more effective behavior and reorganization of self-narrative and identity. The result is the generative and transformational change.

## How the four mechanisms produce clinical change

EFT is experiential and transformative in its processes. In the session the therapist travels with the client on a journey through the client’s emotional landscape. If the session is effective, at the end of 50 min the client’s experience of a traumatic event or a disturbing symptom is radically altered.

The four mechanisms are interactive. They also work in sequence. Reducing dysregulation and reducing emotional avoidance usually create the foundation for change within an EFT powered psychotherapy session, allowing for the emergence of cognitive and emotional restructuring. Together these three mechanisms during the session meet the requirements for the third part of the sequence: reconsolidation.

For instance, a client may use Clinical EFT on a memory of a sexual assault or a war combat trauma that was given a SUDs rating of nine of intensity on scale of 1–10 at the beginning of the session. By the end of the session the SUDS rating may be 0–2. So up to this point, the two most important mechanisms of change are a reduction of dysregulation and reducing emotional avoidance. They work synergistically. The other two mechanism are not required yet.

But additional outcomes also occur, the client’s appraisal of their actions might shift from self-blame and guilt to self-compassion, and this often occurs spontaneously without any reframes by the therapist. This results in the third mechanism, cognitive and emotional restructuring.) This only begins to occur in the context of the first two mechanisms operating. Once the cognitive and emotional restructuring begins to emerge, it loops back and strengthens first two mechanisms in a virtuous cycle.

So, this may be a typical outcome at the end of this session. However, will the changes remain? Long-term follow-up research in many areas of Clinical EFT including PTSD and emotional eating, (1- and 2-year) indicates the changes hold, usually with minimal effort on the part of the client (Church et al., 2016; Stapleton et al., 2012, 2017, 2019). These symptom improvements persist over time, with the treatment effective whether delivered in person or virtually (see Church et al., 2022). The is due to the fourth mechanism of change, reconsolidation. The in-session experiential changes to memories and their associated physiology, affect and meaning are reconsolidated leading to the effortless permanence of the change (Ecker and Vaz, 2022).

The four mechanisms also operate dynamically across clinical presentations. For example, in depression, reducing experiential avoidance allows clients to access underlying emotional pain rather than withdrawing, while cognitive restructuring transforms self-critical narratives into compassionate self-understanding and (memory and schema) reconsolidation updates maladaptive schemas formed in early relational trauma. In anxiety disorders, reducing emotional dysregulation increases tolerance for distressing sensations, interrupting panic escalation, while reducing experiential avoidance directly counters anxiety’s core maintaining factor—the tendency to avoid feared internal experiences. For PTSD, reconsolidation enables traumatic memories to be reprocessed rather than remaining frozen and intrusive, while reducing emotional dysregulation helps clients stay present during trauma processing without dissociating. In building resilience, reducing emotional dysregulation expands capacity to navigate challenges, cognitive restructuring replaces internalized inadequacy with recognition of strengths, and memory reconsolidation updates early attachment-based beliefs about self-efficacy.

## Strength of evidence, limitations, and future directions

While the proposed mechanisms of Clinical EFT are supported by emerging empirical data, the strength of evidence varies across domains. Measures of cortisol reduction (Church et al., 2012; Stapleton et al., 2019) and autonomic nervous system regulation via EEG (Lambrou et al., 2003; Swingle et al., 2004; Swingle, 2010) have the most consistent support, with multiple randomized controlled trials reporting significant improvements in stress regulation and parasympathetic dominance (Church et al., 2022; Feinstein, 2025a, 2025b, 2025c). Neuroimaging evidence showing decreased limbic activation and enhanced prefrontal regulation is promising but limited to small-scale studies (Stapleton et al., 2019, 2023; Wittfoth et al., 2020, 2022). Genetic and epigenetic markers remain nascent, with findings largely theoretical or from pilot studies (Church et al., 2018a, 2018b; Maharaj, 2016).

Key limitations include the predominantly correlational nature of mechanistic studies, leaving causal relationships between tapping and neurobiological changes unresolved, and the absence of longitudinal data on durability of effects. Future research should prioritise multi-modal designs integrating psychophysiological measures, neuroimaging, and clinical endpoints within the same trials. Large-scale, multi-site studies are needed to confirm limbic downregulation and autonomic shifts, while mechanistic trials should explore dose–response relationships and individual differences. Developing unified models that synthesize somatic, cognitive, and neurobiological processes will be critical for advancing EFT toward a fully mechanistic framework.

## Conclusion

This paper demonstrates that Clinical EFT represents a theoretically coherent and empirically grounded psychological intervention. We have articulated a comprehensive model identifying seven active ingredients that work synergistically to engage four core mechanisms of change. This integrative framework positions EFT within established trauma treatment paradigms while highlighting its distinctive contributions, particularly its strategic use of the TOTE model for real-time exposure dosing and its incorporation of somatic intervention for autonomic regulation.

Implications for the field are significant. This model provides a testable framework for mechanistic research, moving beyond correlational studies toward experimental designs that can isolate specific active ingredients. It offers clinicians clearer rationale for treatment selection, particularly for clients who have not responded to exposure-based or cognitive therapies alone. By demonstrating how EFT integrates psychological, somatic, and neurobiological processes, this paper contributes to the broader movement toward transdiagnostic, mechanism-focused interventions in trauma treatment.

Rather than representing an anomalous approach, Clinical EFT exemplifies how innovative therapeutic methods can be rigorously conceptualized within contemporary psychological science. Future research should test and refine this model, examining dose–response relationships, moderators of treatment response, and the durability of neurobiological changes—efforts that will not only strengthen EFT’s empirical foundation but advance our understanding of how multimodal interventions catalyze lasting change in complex trauma.

## Data Availability

The original contributions presented in the study are included in the article/supplementary material, further inquiries can be directed to the corresponding author.

## Footnotes

1 Note this is simply one more TOTE where the final Exit happens only when the entire memory no longer leads to any discomfort.

## Author’s note

Both authors are renumerated for keynotes, workshops, and trainings in clinical EFT due to expertise. PS receives book royalties from Hay House, McFarland Publishers, and Amazon.
